# Increasing Incidence of Invasive *Haemophilus influenzae* Disease in Adults, Utah, USA

**DOI:** 10.3201/eid1709.101991

**Published:** 2011-09

**Authors:** Matthew P. Rubach, Jeffrey M. Bender, Susan Mottice, Kimberly Hanson, Hsin Yi Cindy Weng, Kent Korgenski, Judy A. Daly, Andrew T. Pavia

**Affiliations:** Author affiliations: University of Utah, Salt Lake City, Utah, USA (M.P. Rubach, K. Hanson, H.Y.C. Weng, A.T. Pavia);; Kaiser Permanente, Los Angeles, California, USA (J.M. Bender);; Utah State Department of Health, Salt Lake City (S. Mottice);; Intermountain Healthcare, Salt Lake City (K. Korgenski, J.A. Daly)

**Keywords:** bacteria, vaccine, nontypeable, Haemophilus influenzae, Hia, Hib, Hic, Hid, Hie, Hif, adults, incidence, invasive, Utah, research

## Abstract

Since the introduction of the *Haemophilus influenzae* type b vaccine, the incidence of invasive *H. influenzae* type b disease among children has fallen dramatically, but the effect on invasive *H. influenzae* disease among adults may be more complex. In this population-based study we examined the epidemiology and outcomes of invasive disease caused by typeable and nontypeable *H. influenzae* among Utah adults during 1998–2008. The overall incidence increased over the study period from 0.14/100,000 person-years in 1998 to 1.61/100,000 person-years in 2008. The average incidence in persons >65 years old was 2.74/100,000 person-years, accounting for 51% of cases and 67% of deaths. The incidence was highest for nontypeable *H. influenzae* (0.23/100,000 person-years), followed by *H. influenzae* type f (0.14/100,000 person-years). The case-fatality rate was 22%. The incidence of invasive *H. influenzae* in Utah adults appears to be increasing. Invasive *H. influenzae* infection disproportionately affected the elderly and was associated with a high mortality rate.

## Abstract 2

TOC Summary: The infection disproportionately affected patients >65 years of age.

Before the introduction of the *Haemophilus influenzae* type b (Hib) conjugate vaccine, Hib was the most common cause of bacterial meningitis in children <5 years of age (*1*,*2*). Hib caused >80% of invasive *H. influenzae* disease among children (*3*). Since the introduction of the Hib conjugate vaccine, the incidence of invasive Hib disease in children has decreased by 99% (*1*,*2*). Several studies have reported a decrease in the incidence of invasive Hib in adults following widespread use of vaccine in children (*4*,*5*).

However, although the decrease in the overall incidence of invasive disease caused by Hib for children and adults has been a remarkable public health success, changes in the epidemiology of invasive disease caused by other *H. influenzae* have been observed. The incidence of *H. influenzae* invasive disease has shifted toward adults. In a study conducted in the Atlanta, Georgia, USA, metropolitan area before Hib vaccine introduction, adults comprised 24% of all invasive *H. influenzae* cases (*6*). A more recent population-based report from Illinois showed adults accounting for 77% of invasive cases (*7*). Other encapsulated strains, including *H. influenzae* type a (Hia), have emerged as notable causes of invasive disease among children in Utah, Alaska, and the Navajo nation (*8*–*10*). Given the changing epidemiology observed elsewhere, we sought to describe the epidemiology of invasive *H. influenzae* infections among Utah adults during 1998 through 2008.

## Methods

### Study Design and Population

We identified all cases of laboratory-confirmed invasive *H. influenzae* among Utah residents >18 years of age reported to the Utah Department of Health (UDoH) from 1998 through 2008. Invasive *H. influenzae* is an immediately reportable condition in Utah. We defined invasive *H. influenzae* according to the Council of State and Territorial Epidemiologists definition: a clinically compatible case that is confirmed by isolation of *H. influenzae* from a normally sterile site such as blood, cerebrospinal fluid, joint fluid, pleural fluid, or pericardial fluid (*11*). The UDoH enhanced their passive surveillance program in 2005 by sending out reminders to major laboratories in Utah to report invasive *H. influenzae* cases. As part of these enhancement efforts, 1 large laboratory also began automated case reporting to the UDoH in 2005. Because these changes in surveillance could introduce ascertainment bias for the final 4 years of this analysis, we conducted a parallel retrospective cohort analysis of laboratory-confirmed invasive *H. influenzae* cases among Utah residents within the Intermountain Healthcare (IH) system during 1999–2008 (on the basis of availability of complete laboratory data). IH is a vertically integrated hospital system that provides care to roughly 50% of Utah adults (Scott Lloyd, pers. comm.). The estimated market share by age group is recalculated at regular intervals. Over this period, IH made no major changes to its microbiology practices that would alter the recovery of *H*. *influenza* isolates.

### Serotyping

*H. influenzae* isolated from sterile sites was identified by using standard culture techniques. Laboratories are requested to submit sterile site isolates of *H. influenzae* to the Utah Public Health Laboratory in Salt Lake City, Utah, for definitive serotyping by using monovalent antisera for *H. influenzae* types a–f. Isolates from reported *H. influenzae* cases not sent to the Utah Public Health Laboratory for serotyping were classified as not typed. All other isolates were classified as Hia–f or as nontypeable.

### Demographic and Outcome Data

As part of its surveillance program, the UDoH collected demographic information including county of residence, race/ethnicity, age, gender, hospitalization, and death. Starting in 2005, the UDoH began to collect expanded clinical information including the presence of chronic medical conditions and clinical signs. These clinical data were collected at the local health department level. It should be noted that not all local health departments conducted such data collection, and collection methods varied across local health departments (e.g., chart review, patient interview, hospital information personnel interview).

### Statistical Analysis

To calculate the incidence rate, we used the estimated annual Utah population among persons >18 years of age on the basis of the United States 2000 census (*12*). To derive an incidence for the IH cohort, we multiplied the estimated annual population by IH estimated market share of adult healthcare in Utah for a given year. Market share data were only available for 2000, 2002, and 2005–2008. For intervening years, we extrapolated using available data for the nearest years.

Categorical variables were evaluated by using standard χ^2^ test for independence or Fisher exact test as appropriate. To evaluate associations between variables, we used ordinary logistic regression and exact logistic regression as appropriate. Poisson regression method with a log-linear model was performed to test for trend variation in the annual case rates for the IHC and UDoH datasets. We used SAS version 9.2 (SAS Institute Inc., Cary, NC, USA) for all statistical analyses.

### Human Subjects Protection

We obtained approval for this study from the Institutional Review Boards of the University of Utah and IH; de-identified case information was provided by the UDoH through a data sharing agreement. Informed consent was waived as all data were de-identified.

## Results

**Figure 1:**
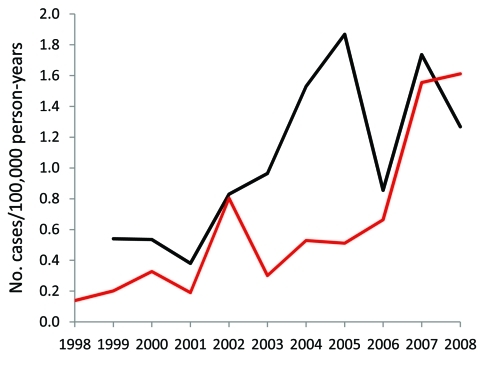
Comparison of annual incidence of invasive *Haemophilus influenzae* disease derived from the Utah Department of Health and Intermountain Healthcare databases, Utah, USA, 1998–2008. Black line, Intermountain Healthcare; red line, Utah Department of Health.

We identified 121 cases of invasive *H. influenzae* in Utah adults reported to the UDoH during 1998–2008 (Table). The incidence of *H. influenzae* invasive disease was 0.66 cases per 100,000 person-years averaged over the 11 years. Incidence increased over the study period from 0.14 cases per 100,000 person-years in 1998 to a peak in 2008 of 1.61 cases per 100,000 person-years (p = 0.0023, by linear test for trend) (Figure 1). We observed a marked seasonality with winter predominance: 41.3% of cases occurring in winter, 26.4% in spring, 21.4% in fall, and 10.7% in summer. We observed no significant difference in distribution between sexes, with 64 cases in women and 57 cases in men (p = 0.65).

**Table:** Characteristics, by serotype, of 121 cases of invasive *Haemophilus influenzae* in adult patients, Utah, USA, 1998–2008

Characteristic	No. (%) samples								
	Type a, n = 15	Type b, n = 9	Type c, n = 1	Type d, n = 3	Type e, n = 5	Type f, n = 25	Not typeable, n = 43	Not typed, n = 20	Total
Patient age, y									
18–34	3 (20.0)	0	1	0	0	1 (4.0)	9 (20.9)	3 (15.0)	17 (14.0)
35–49	3 (20.0)	3 (33.3)	0	0	0	1 (4.0)	5 (11.6)	1 (5.0)	13 (10.7)
50–64	4 (26.7)	2 (22.2)	0	1	1	6 (24.0)	12 (27.9)	3 (15.0)	29 (23.9)
>65	5 (33.3)	4 (44.4)	0	2	4	17 (68.0)	17 (39.5)	13 (65.0)	62 (51.2)
Patient sex, F	8 (53.3)	4 (44.4)	1	0	3	17 (68.0)	21 (48.8)	10 (50.0)	64 (52.8)
Sample source									
Cerebrospinal fluid	1 (6.7)	4 (44.4)	1	0	0	4 (16.0)	5 (11.6)	0	15 (12.3)
Blood	14 (93.3)	5 (55.6)	0	3	5	21 (84.0)	35 (81.3)	18 (90.0)	101 (83.5)
Other	0	0	0	0	0	0	3 (6.9)	2 (10.0)	5 (4.1)
Patient death	4 (26.7)	2 (22.2)	0	1	0	4 (16.0)	10 (23.2)	6 (30.0)	27 (22.3)

The incidence rates estimated by using the IH data showed a similar trend (Figure 1). We identified 94 cases of invasive *H. influenzae*, with an estimated average annual incidence of 1.05/100,000 person-years. The incidence increased from 0.54/100,000 person-years in 1999 to 1.27/100,000 person-years in 2008 (p = 0.002, by linear test for trend). Although the estimated rates differed, the trend in increasing incidence was similar for both data sources.

**Figure 2:**
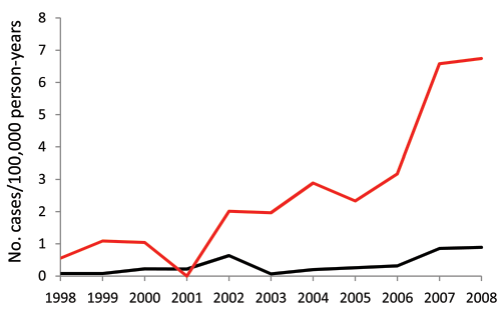
Annual incidence of invasive *Haemophilus influenzae* disease in adults, by age, Utah, USA, 1998–2008. Red line, age >65 y; black line, ages 18–64 y.

The incidence of invasive disease was markedly higher among persons >65 years of age; the average incidence in this age group was 2.74 cases per 100,000 person-years and increased to 6.14 cases per 100,000 in 2008. The average incidence for persons 18–34, 35–49, and 50–64 years of age were 0.25, 0.26, and 0.88 per 100,000 person-years, respectively. Compared with persons 18–34 years of age, patients >65 years of age had a 12-fold greater risk for invasive disease (risk ratio 12.5, 95% confidence interval [CI] 7.29–21.32; p<0.0001). The incidence of invasive disease increased significantly over the study period among those >65 years of age (Figure 2).

### Serotype Distribution

**Figure 3:**
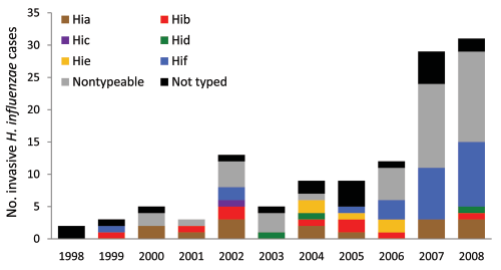
No. cases of invasive *Haemophilus influenzae* (Hi) disease in adults, by serotype (a–f), Utah, USA, 1998–2008.

Of the 121 patients with invasive disease, 101 (83%) isolates were serotyped. Figure 3 depicts the annual number of cases by serotype and isolates that did not undergo serotype testing. Nontypeable strains accounted for 43 cases (43%). Hib accounted for 9 cases (9%) and non-b encapsulated strains accounted for 49 cases (49%). Serotypes f and a accounted for 25 (25%) and 15 cases (15%), respectively.

The increases in invasive disease were largely because of nontypeable strains and Hif. The average incidence over 11 years of invasive disease caused by nontypeable strains was 0.23/ 100,000 person-years, and rose from 0 in 1998 and 1999 to an average of 0.72/100,000 person-years during 2007 and 2008. During 1998–2008, the average incidence of disease caused by Hif was 0.14/100,000 person-years and rose to an average of 0.48/100,000 person-years during 2007 and 2008. The number of cases of Hia remained relatively steady, with an average annual incidence of 0.08/100,000 person-years.

### Clinical Disease

Most (51%) invasive disease occurred in persons >65 years of age. Bacteremia accounted for 83% of all cases, meningitis for 12.4%, and other sterile sites for the remaining 4.6%. Nontypeable strains, Hib, and Hif accounted for 13 of the 15 cases of meningitis (Table). Infection with Hib was significantly more likely to be associated with meningitis than nontypeable strains (odds ratio [OR] 6.1, 95% CI 1.3–29.0). While invasive disease was more likely to occur in the elderly, a greater proportion of disease among patients 18–49 years of age was due to meningitis compared with patients >50 years of age (25% vs. 9%; p = 0.05).

Data on underlying illness was available for 69 of the 121 case-patients. Forty-six (67%) of 69 patients had chronic medical conditions; 33% had >2. Twenty-four patients had diabetes mellitus; 15 were immunocompromised; 14 had chronic lung disease; 11 had a malignancy; 3 had asthma; 3 each had chronic obstructive pulmonary disease, congestive heart failure, chronic liver disease/cirrhosis, and chronic kidney disease; and 1 had a history of intravenous drug use. Forty-six patients had a diagnosis of pneumonia at the time of *H. influenzae* isolation. Patients with pneumonia were significantly more likely to have >1 chronic illness (OR 4.48, 95% CI 1.48–13.58, p = 0.008) and significantly less likely to have meningitis (OR 0.048, 95% CI 0–0.358, p = 0.0016).

The overall case-fatality rate was 22%. Two thirds of deaths were among those >65 years of age. Persons >65 years of age had a case-fatality rate of 29% compared to 15% for those <65 years of age (p = 0.08). The case-fatality rate for patients with bacteremia (23%) was not significantly higher than for patients with meningitis (13%). The case-fatality rate did not differ significantly by serotype. Outcome did not differ significantly by the presence or absence of concurrent conditions (17% vs. 26% mortality rate, respectively), but data were only available for 69 patients.

## Discussion

Despite the virtual elimination of Hib in areas with widespread use of Hib vaccine, invasive disease caused by *H. influenzae* remains a major source of illness and death. The incidence of invasive *H. influenzae* in Utah adults appears to be increasing; most of the increase in *H. influenzae* disease incidence was attributable to an increase in nontypeable and Hif strains. We found that invasive *H. influenzae* disease was most common in persons >65 years of age and was associated with a high mortality rate.

The overall incidence of invasive *H. influenzae* disease in adults in our population, 0.66 cases per 100,000 person-years, was similar to rates observed in Illinois (1.0/100,000 person-years in 1994) (*7*) and slightly lower than rates among Alaskans during 1991–1996 (1.4/100,000 person-years) (*5*). We found a significant increase in the incidence of invasive *H. influenzae* over the study period by using 2 surveillance methods. Several studies in addition to ours suggest an increase in invasive disease caused by to non-type b *H. influenzae* among adults (*5*,*7*,*13*). The reasons for this change are unclear, and might reflect changes in the organisms, changes in the number of persons at high risk, or perhaps waning of cross-immunity induced by exposure to Hib.

As the prevalence of Hib has decreased, other encapsulated serotypes seem to have emerged as major causes of invasive disease, including Hif in Illinois and Hia in Brazil, Manitoba, and Northwestern Ontario (*7*,*10*,*14*,*15*). Similar to findings in Illinois, Hif was the second most common serotype in our analysis (25%), and cases of invasive Hif contributed substantially to the increase of invasive *H. influenzae* cases we observed in 2007–2008. Although Hia was the third most common isolate among adults in our study, it accounted for only 15% of all cases, a rate that is much lower than rates in Manitoba (29%) (*15*), Northwestern Ontario (42%) (*10*), and in children in Utah (*8*).

Nontypeable *H. influenzae* has been the most common isolate in virtually all published series among adults. In our study, it accounted for 43% of cases; similar rates were reported in other studies (*3*,*7*,*15*–*17*). The emerging role of invasive disease because of nontypeable strains is intriguing because this organism has traditionally been considered a relatively noninvasive bacteria predominantly associated with community-acquired pneumonia, chronic obstructive pulmonary disease exacerbations, and otitis media (*18*). Whether invasive strains of nontypeable *H. influenzae* have distinct genotypic and phenotypic characteristics compared with noninvasive isolates remains largely unknown. Candidate virulence factors include the adhesin genes *hmw* and *hia* and IS*1016*, an insertion element that may confer Hib-like, encapsulated properties to nontypeable strains. However, to date no single set of virulence determinants has been conclusively associated with invasive nontypeable strains (*19*–*21*). Better understanding of the factors that confer invasive capabilities might lead to improved strategies for vaccine development.

Similar to the findings in Spain, Illinois, and Alaska, rates of disease in our study were highest among older patients (*3*,*5*,*7*). The incidence increased in persons >65 years of age, and 29% of infections in this age group were fatal, emphasizing the disproportionate effect. Invasive *H. influenzae* was highly associated with concurrent illness. Among those for whom clinical data were available, 67% of our patients had an underlying condition, as has been observed in other studies (*3*,*7*). However, in those with and without concurrent illness, invasive *H. influenzae* showed a high overall case-fatality rate (22%). Other studies have reported that invasive *H. influenzae* is associated with high mortality rates, ranging from 13% to 29% (*3*,*4*,*6*,*7*). During 2005–2008, the CDC Active Bacterial Core surveillance reported case-fatality rates of 13%–17% for invasive *H. influenzae* (*22*). Among adults >65 years of age, the incidence of invasive *H. influenzae*, the case-fatality rate and the number of attributable deaths exceeds that for invasive meningococcal disease in the most vulnerable age group, those 11–19 years of age (*23*). However, no vaccine is available for invasive disease caused by encapsulated non-type b and nontypeable *H. influenzae*. Efforts to prevent invasive *H. influenzae*, particularly among the elderly, should become a public health priority.

As with all surveillance studies, our study is subject to several limitations. The UDoH relies on passive reporting of *H. influenzae* from laboratories throughout the state, making underreporting likely. However, a study of invasive *H. influenzae* in Spain among children <5 years of age demonstrated that passive surveillance had a sensitivity of 88% (*24*). Comparison of estimated rates from the IH laboratory database and UDoH surveillance data suggests that the sensitivity of reporting among adults was 65%. Changes in reporting behavior and changes to the passive surveillance program in 2005 are potential sources of bias. Several studies have demonstrated the inaccuracy of serotyping compared with molecular diagnostic techniques (*25*,*26*). Although serotyping is still an accepted standard, had we performed capsular gene analysis, it might have changed the serotype distribution. Additionally, the small number of identified cases places limitations on analyses of serotype distribution and clinical characteristics. Finally, we had clinical information on concurrent conditions for only 57% of the cohort, and clinical information was not collected in a systematic manner across the state.

These limitations notwithstanding, we found that the incidence of invasive *H. influenzae* in Utah adults appears to be increasing, and this increase is caused mostly by the rising incidence of nontypeable *H. influenzae* and Hif. Invasive *H. influenzae*, including disease caused by nontypeable strains, has a high mortality rate. Persons >65 years of age are most affected by the disease and have the highest death rates. These data have implications for targeted adult *H. influenzae* vaccine development.
